# Thermal Characterization of Crosslinked Polymeric Microspheres Bearing Thiol Groups Studied by TG/FTIR/DSC under Non-Oxidative Conditions

**DOI:** 10.3390/ma17061372

**Published:** 2024-03-17

**Authors:** Magdalena Maciejewska, Elżbieta Łastawiecka, Marta Grochowicz

**Affiliations:** 1 Department of Polymer Chemistry, Institute of Chemical Sciences, Faculty of Chemistry, Maria Curie-Sklodowska University, 20-614 Lublin, Poland; magdalena.maciejewska@mail.umcs.pl; 2 Department of Organic Chemistry and Crystal Chemistry, Institute of Chemical Sciences, Faculty of Chemistry, Maria Curie-Sklodowska University, 20-614 Lublin, Poland; elzbieta.lastawiecka@mail.umcs.pl

**Keywords:** polymer microspheres, thermal stability, thiol group, thermal characteristic

## Abstract

This paper presents the thermal behavior of polymer microspheres based on glycidyl methacrylate (GMA) and crosslinking agents benzene-1,4-diylbis(2-methylprop-2-enoate) (1,4DMB) and trimethylolpropane trimethacrylate (TRIM) before and after functionalization with thioglycolic acid (TGA). The thermal stability of the polymers was determined using thermogravimetric analysis and differential scanning calorimetry under non-oxidizing conditions. The evolved gases were detected by FTIR and NMR spectroscopy, and the chemical structure of solid residues after preheating was assessed by FTIR/ATR spectroscopy. The post-functionalized microspheres showed higher thermal stability (within 270–290 °C) than the initial copolymers (within 240–250 °C). In this paper, examples of decomposition patterns of polymer microspheres before and after functionalization are presented. The decomposition of the initial microspheres starts with the emission of GMA monomers, acrolein, carbon dioxide, and the formation of unsaturated bonds in the solid residue. In the case of functionalized microspheres, degradation involves the transesterification of ester groups with the -SH groups, resulting in the emission of carbonyl sulfide, acrolein and carbon dioxide. Furthermore, lactone groups are created in the solid residue. The degradation of the functionalized copolymers is a complex process due to their crosslinked structure, rendering the identification of all the degradation products unattainable.

## 1. Introduction

Porous crosslinked polymers in the form of microspheres based on vinyl monomers with reactive functional groups in their structure can be easily functionalized. Among these vinyl monomers, glycidyl methacrylate (GMA) stands out due to its dual functionality from both methacrylate and oxirane groups. Its chemical structure enables flexibility in designing the polymer structure [1,2,3,4,5,6]. The methacrylate group contains a reactive C=C bond that allows it to polymerize readily with a wide range of functional and crosslinked monomers. Such monomers include aliphatic methacrylates, like ethylene glycol dimethacrylate, trimethylolpropane trimethacrylate (TRIM), or aromatic monomers, like the widely used divinylbenzene (DVB) [6,7,8,9,10,11]. On the other hand, the oxirane group introduced to the polymer structure by GMA can react with strong and weak nucleophiles, e.g., amines [12,13,14,15], which are reagents that allow the introduction of carboxyl groups [16,17], pyrrolidone [18,19] and many others. The modification with a reactive oxirane group is more demanding, but some functional groups, like a thiol group (-SH), can only be introduced into the microsphere structure in this way. Polymer microspheres that contain thiol groups within their structure are attractive materials for a variety of specialized applications. Some examples include resins for heavy metal sorption [20,21,22], sorbents for column packings for chromatography and solid phase extraction [23,24,25,26], supports for solid-phase organic synthesis in the preparation of oligopeptides or various monosaccharide derivatives, substrates for the heterogenous catalysts used in organic chemistry [27] and matrices for nanocomposite synthesis with metals like silver, gold or quantum dots [28,29,30].

The incorporation of thiol groups into the structure of polymer microspheres has been reported in the literature most often as a multi-stage process. Since it involves the presence of a halomethyl group, polymer networks containing aromatic rings susceptible to electrophilic aromatic substitution are usually preferable. The halomethyl groups undergo the reaction with a suitable sulfur compound giving an intermediate product that must be hydrolyzed in a subsequent step to obtain the thiol group [21,22,31,32,33]. The described methods are time-consuming, the reactions involved must be carried out with catalysts and in organic solvents, and often involve harsh conditions for porous microspheres, which can destroy their porous structure or even the entire microsphere. In our previous paper [34], a new method for the modification of polymer microspheres with thiol groups was presented. Crosslinked, porous GMA-based copolymer microspheres possessing thiol groups in their chemical structure were obtained in the reaction with thiocarboxylic acids in a one-step process in a short time and in a catalyst- and solvent-free environment. These reaction conditions preserve the permanent internal porosity and the spherical structure of the polymer particles.

We also reported [34] the thermal properties of these new copolymers studied in an oxidative atmosphere. The purpose of the present investigation was to characterize the thermal behavior of porous copolymer microspheres of GMA crosslinked with benzene-1,4-diylbis(2-methylprop-2-enoate) (1,4DMB), (poly(GMA-*co*-1,4DMB)) and GMA crosslinked with TRIM (poly(GMA-*co*-TRIM)) and their derivatives bearing thiol groups in non-oxidative conditions. To our knowledge, the results of the behavior of these copolymers during pyrolysis have not been reported in the literature. However, it is an important issue, especially in terms of copolymer recycling and possible reuse of polymeric sorbents. In this study, an analysis was conducted before and after the modification of microspheres with thioglycolic acid using thermogravimetric analysis (TG) coupled with FTIR spectroscopy, differential scanning calorimetry (DSC) and nuclear magnetic resonance (NMR). The use of NMR as a tool for the detection of volatiles is a novel approach enabling more complete identification of polymer degradation products. Additionally, the thermal degradation pathways of the starting and thiol-functionalized microspheres are presented based on their degradation products.

## 2. Materials and Methods

### 2.1. Materials

The monomers used for polymer synthesis, including styrene (99%), glycidyl methacrylate (GMA; 97%) and trimethylolpropane trimethacrylate (TRIM), thioglycolic acid (TGA; 98%) used for functionalization reactions, 2,2′-azobis(2-methylpropionitrile) (AIBN; 98%) used as an initiator of polymerization, polyvinylpyrrolidone (PVP40, average molecular mass of 40,000 g/mol) and sodium lauryl sulfate (SDS; 99%) used as polymerization medium were purchased from Sigma-Aldrich. Toluene, tetrahydrofuran (THF), acetone, methanol and ethanol were obtained from POCh (Gliwice, Poland). Benzene-1,4-diylbis(2-methylprop-2-enoate) (1,4DMB) was obtained according to the previously described procedure [24].

### 2.2. Synthesis of Porous Polymer Microspheres

Porous crosslinked microspheres were prepared using seed swelling polymerization according to a previously described procedure [34]. Firstly, polystyrene seed microspheres (PS) with a diameter of about 2 µm were obtained via dispersion polymerization in ethanol/PVP solution. Then, PS seeds were dispersed in aqueous solution of SDS and a mixture of GMA, 1,4DMB or TRIM (with a molar ratio of GMA to crosslinking monomers of 3:1), AIBN and toluene (acting as a porogen). The dispersion prepared in this way was left for 24 h under stirring (100 rpm) to absorb the monomers -into the PS seeds. In the next step, the temperature was increased to 71 °C and polymerization was carried out for 24 h under continuous stirring (100 rpm). After the reaction, the microspheres were filtered, washed with hot distilled water and acetone, respectively, and extracted with the use of THF under reflux for 6 h with constant stirring (250 rpm). Finally, the microspheres were filtered, washed with THF, and dried under room conditions. In this procedure, two types of polymer microspheres were obtained, denoted as poly(GMA-*co*-1,4DMB) and poly(GMA-*co*-TRIM).

**Figure 1:**
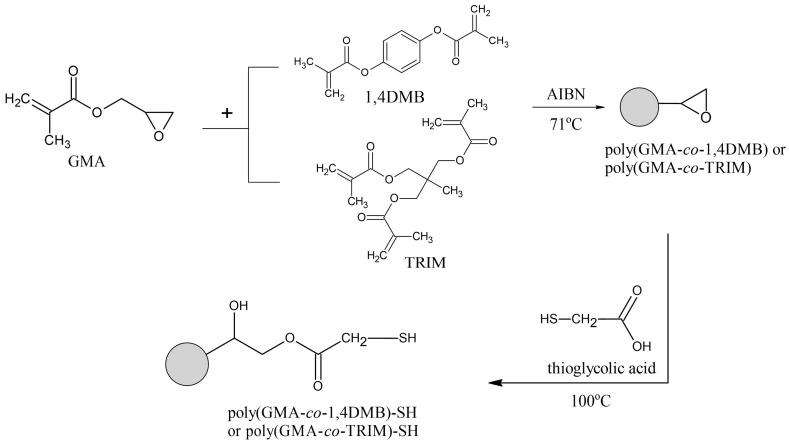
General scheme of copolymeric microsphere synthesis.

The oxirane ring opening reaction was carried out as shown in Figure 1, according to a previously described procedure [34]. To achieve this, a mixture of microspheres and thioglycolic acid in a volume ratio of 1:1.5 was placed in a test tube and closed with a Teflon plug. The reaction was carried out in a microwave reactor at 100 °C for 4 min with constant stirring. After cooling to room temperature, the reaction mixture was centrifuged. The supernatant containing unreacted acid was decanted. The microspheres were then extracted with acetone and methanol to remove any remaining acid, and finally dried under room conditions. As products of the functionalization, two types of microspheres were synthesized and denoted as poly(GMA-*co*-1,4DMB)-SH and poly(GMA-*co*-TRIM)-SH.

### 2.3. Material Characterization

The chemical structure of synthesized polymers was assessed using the FTIR method with the use of a Tensor 27 FTIR spectrometer (Bruker, Mannheim, Germany) with the ATR technique. The ATR accessory was equipped with a diamond crystal. The spectra were recorded in the range of 600–4000 cm^−1^ with a resolution of 4 cm^−1^. In total, 16 scans were made for each spectrum.

Thermal analysis was conducted with the use of an STA 449 F1 Jupiter thermal analyzer (Netzsch, Selb, Germany) in a helium atmosphere from 35 to 850 °C. The gas flow rate was 25 mL/min. The heating rate was 10 °C/min. Samples weighing about 10 mg were tested in open Al_2_O_3_ crucibles. An empty Al_2_O_3_ crucible was used as a reference. The gaseous products of polymer decomposition were passed directly from an STA instrument to the FTIR analyzer (FTIR TGA 858, Bruker, Germany). Spectra were recorded every 10 °C (16 scans) in the range of 4000–600 cm^−1^ with a 4 cm^−1^ resolution. Moreover, the gaseous products resulting from the degradation of thiol-containing polymers were analyzed by ^1^H NMR. The samples for analysis were collected directly from the STA analyzer. To collect the gases, a short Teflon tube was used to lead the gases out of the STA into a glass test tube containing 1 mL of CDCl_3_ cooled to −40 °C (acetonitrile/dry ice bath). Gases were collected in the solvent from the starting temperature to 390 °C. The ^1^H NMR spectra of trapped products were obtained with the use of a Bruker Ascend (500 MHz) spectrometer at room temperature.

Differential scanning calorimetry measurements were performed using a DSC 204 calorimeter (Netzsch, Germany) operating in dynamic mode. DSC scans were taken in the following three cycles: heating from room temperature to 150 °C (to remove any adsorbed moisture), cooling from 150 °C to 20 °C and heating from 20 °C to 500 °C with a heating/cooling rate of 10 °C/min. Measurements were taken in argon with a flow rate of 30 mL/min. Samples weighing about 10 g were tested in pierced aluminum crucibles.

## 3. Results and Discussion

The subject of the current study is the characterization of the thermal behavior under pyrolysis conditions of polymer microspheres containing oxirane rings or thiol groups in their structure. Poly(GMA-*co*-TRIM) and poly(GMA-*co*-1,4DMB) microspheres were synthesized using seed swelling polymerization with GMA functional monomer and the following crosslinking agents: TRIM and 1,4DMB. The synthesized microspheres were subsequently modified with thioglycolic acid to introduce thiol groups on their surface. The epoxide number of starting copolymers was determined using HCl/dioxane reverse titration. The value obtained was 1.23 mmol/g for poly(GMA-*co*-TRIM) and 1.12 mmol/g for poly(GMA-*co*-1,4DMB). The sulfur content of the copolymers after functionalization, determined through elemental analysis, was 3.12% and 4.51% for poly(GMA-*co*-TRIM)-SH and poly(GMA*-co-*1,4DMB)-SH, respectively.

**Figure 2:**
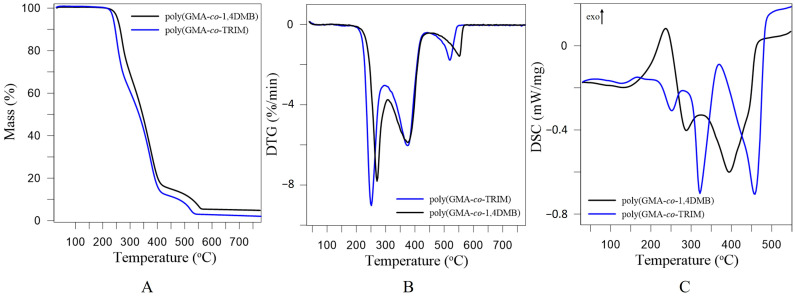
TG (**A**), DTG (**B**) and DSC (**C**) curves of poly(GMA*-co-*1,4DMB) and poly(GMA*-co-*TRIM) copolymers obtained under non-oxidative conditions.

The thermal properties of the studied copolymers were characterized based on the results obtained from thermogravimetric analysis and differential scanning calorimetry. In Figure 2, TG, DTG and DSC curves for poly(GMA-*co*-1,4DMB) and poly(GMA-*co*-TRIM) are shown. Table 1 presents the temperatures of 5% and 50% mass loss (T_5%_, T_50%_), the temperatures of maximum mass loss (T_max_) and the corresponding mass loss (Δm). The DTG curves indicate that the starting copolymers undergo a three-step degradation process in an inert atmosphere, regardless of the crosslinking agent used. Poly(GMA-*co*-TRIM) shows the first maximum value of degradation at 251 °C and is accompanied by a mass loss of 39.4% (Δm_1_). The second maximum value occurs at 375 °C with a mass loss of 49.3% (Δm_2_). The third maximum value is observed at 519 °C, with a mass loss of 9.6% (Δm_3_). The DSC curve reveals three distinct maxima that correspond to the maxima on the DTG curve, indicating that the various stages of copolymer degradation are overall endothermic processes. Based on the T_5%_ value, it can be concluded that poly(GMA-*co*-TRIM) is stable up to 238 °C under non-oxidative conditions. In contrast, the thermal stability of poly(GMA-*co*-1,4DMB) copolymers is higher by about 15 °C in comparison with the TRIM-crosslinked material. The first stage of poly(GMA-*co*-1,4DMB) decomposition occurs at 269 °C and is connected with a mass loss of 33.8% (Δm_1_). The second stage occurs at 388 °C with a mass loss of 49.9% (Δm_2_), and the third at 556 °C with a mass loss of 11.2% (Δm_3_). The course of TG curves for both copolymers is similar to the previously reported porous microspheres crosslinked with 1,4DMB or TRIM analyzed in a non-oxidative atmosphere [13,35]. The course of the DSC curve of poly(GMA-*co*-1,4DMB) differs in comparison with the TRIM-crosslinked copolymer. In the temperature region between 100 and 250 °C, the exothermic peak evolving into an endothermic peak is observed for this copolymer, while the next peak is endothermic.

**Figure 3:**
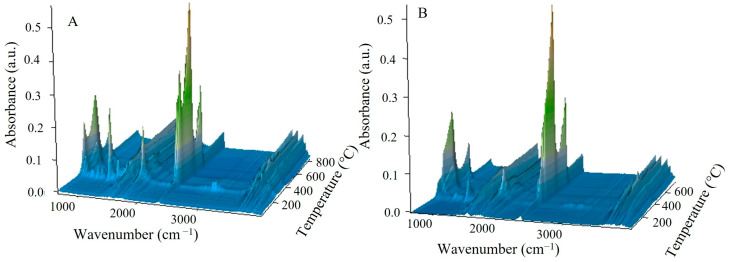
Three-dimensional FTIR diagrams of gaseous products of poly(GMA-*co*-TRIM) (**A**) and poly(GMA-*co*-1,4DMB) (**B**) degradation.

**Figure 4:**
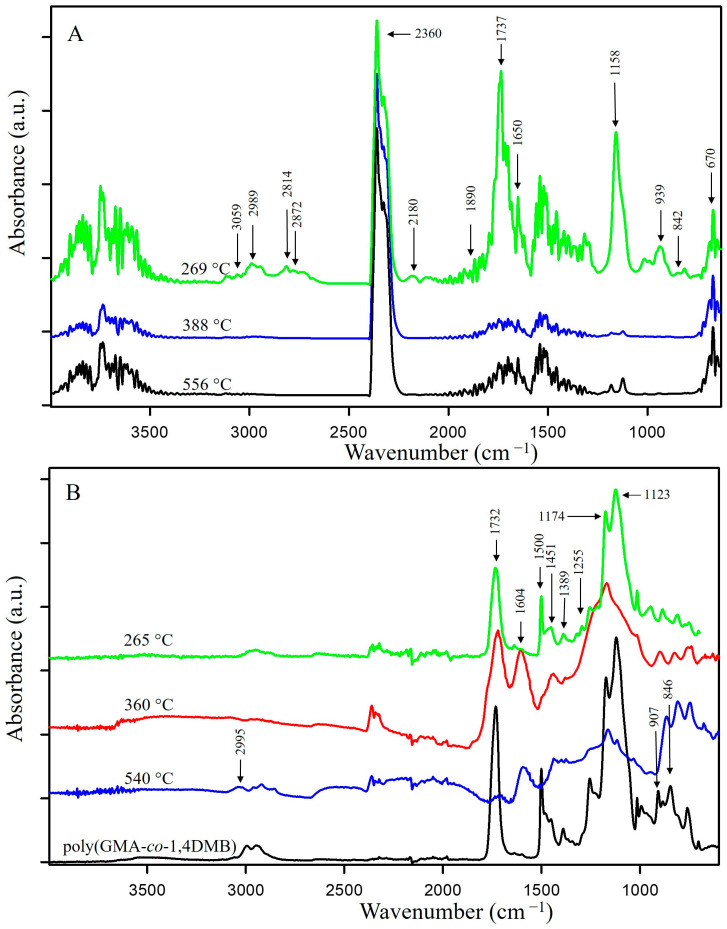
FTIR spectra of (**A**) gases released from poly(GMA-*co*-1,4DMB) at the maxima of emission; (**B**) preheated poly(GMA-*co*-1,4DMB).

**Figure 5:**
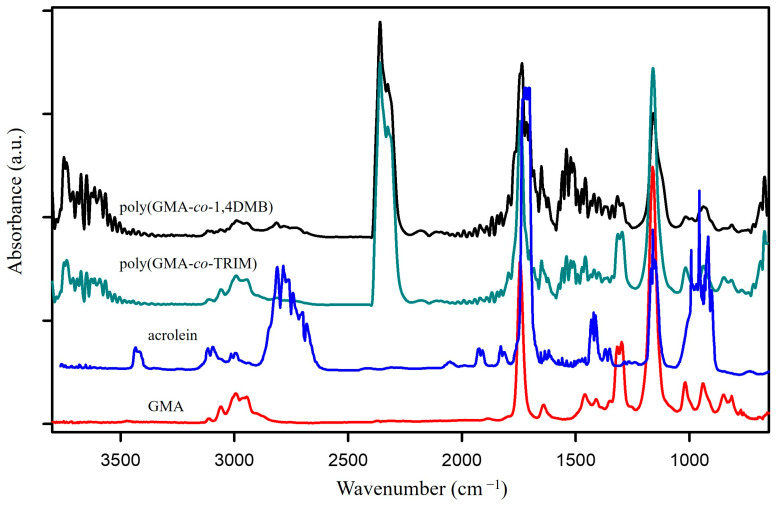
FTIR spectra of gaseous decomposition products of poly(GMA-*co*-1,4DMB) at 269 °C and the reference spectra of GMA monomers and acrolein.

**Figure 6:**
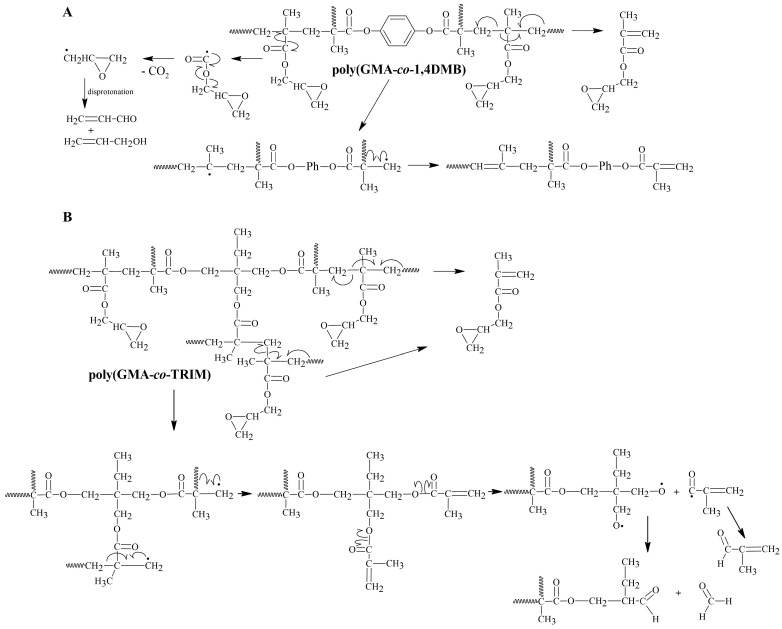
The possible decomposition path of the 1,4DMB crosslinked copolymer (**A**) and TRIM crosslinked copolymer (**B**).

The explanation for this overall exothermic peak should be found in the mechanism of the degradation of the copolymer in a non-oxidizing atmosphere. According to literature sources, the thermal degradation of the polyGMA homopolymer starts with endothermic depolymerization [36] followed by exothermic isomerization of the oxirane ring to the carbonyl group [16,37]. In the case of poly(GMA-*co*-1,4DMB), decomposition is also triggered by endothermic depolymerization but it is a complex process with overlapping reactions, including the exothermic isomerization of oxirane. Additionally, the mass loss at the first degradation step is significant and shifts the DSC signal in the exothermic direction. Hence, the first signal observed on the DSC curve is exothermic as a result of various reactions occurring simultaneously [38], but it should be noted that the decomposition is initially endothermic. To confirm the mechanism of copolymer decomposition, FTIR analysis of the emitted volatile degradation products was performed. Based on the 3D FTIR spectrum (Figure 3) and the spectra extracted at maximum degradation temperatures (Figure 4A), it can be concluded that the depolymerization occurs at the beginning of poly(GMA-*co*-1,4DMB) microsphere decay and is followed by isomerization in the gas phase. The FTIR spectrum of gases gathered at 269 °C reveals the following bands: bands at 3059 cm^−1^ associated with vibrations of the methylene group in the oxirane ring [39]; bands at 1737 cm^−1^ from the vibrations of the C=O group in esters [40]; bands at 1650 cm^−1^ associated with vibrations of the C=C bond of the methacrylate group; bands at 1158 cm^−1^ related to vibrations of the C-O-C group in esters; bands at 939 cm^−1^ derived from vibrations of the =CH_2_ group; bands at 842 cm^−1^ due to vibrations of the oxirane ring [41]. These bands are characteristic of GMA monomer [42] and their presence testifies that depolymerization takes place at the beginning of the decomposition. Furthermore, the FTIR spectrum of gaseous degradation products released at 269 °C exhibits bands at 2814, 2782 and 1890 cm^−1^ that are characteristic of acrolein, which is a product of ester bond breakdown of gaseous GMA and subsequent isomerization [16]. Figure 5 shows FTIR spectra of the gaseous products of copolymer decomposition at 269 °C, along with the reference spectra of GMA monomers and acrolein obtained from the NIST database [42,43]. Moreover, from the spectrum recorded at 269 °C, it is visible that the emission of carbon dioxide (2360 and 670 cm^−1^), carbon monoxide (2180 cm^−1^) and water (3600–3500 cm^−1^) also take places in the first stage of pyrolysis [40]. These inorganic species were formed as a result of dehydration and decarboxylation of the radical intermediate pyrolysis products [44]. To confirm the decomposition course of poly(GMA-*co*-1,4DMB), the material was heated to 265, 360 and 540 °C, respectively, in a helium atmosphere, and FTIR/ATR spectra of the preheated copolymers were then recorded. The spectra of the initial copolymer and the samples heated to higher temperatures are shown in Figure 4B. The disappearance of the bands at 846 and 907 cm^−1^ derived from the vibrations of the oxirane ring can be observed in the spectrum of the sample heated to 265 °C, compared to the initial spectrum [41]. Furthermore, a band at 1255 cm^−1^ representing the stretching vibrations of the oxirane group also disappears [45]. The lack of the absorption bands originating from the oxirane groups in this spectrum confirms that the depolymerization and emission of the GMA monomer occur in the first stage of copolymer degradation. In the spectrum of the copolymer heated to 360 °C, there is no band at 1500 cm^−1^ originating from C=C vibrations of the aromatic ring [46]. The disappearance of bands at 1174 and 1123 cm^−1^ originating from -C-O-C- stretching vibrations in the ester can also be observed. Simultaneously, a sharp band with a maximum at 1168 cm^−1^ appears and a shift of the C=O group band from 1732 to 1720 cm^−1^ and a decrease in its intensity take place, indicating partial decarboxylation (CO_2_ emission) and the formation of an aldehyde group. In addition, a band at 1604 cm^−1^ originating from the C=C vibration can be observed [47]. The disappearance of bands at 1451, 1389 and 2995 cm^−1^ associated with vibrations of methyl and methylene groups is also visible [48]. Based on the obtained spectroscopic results, a mechanism for the decomposition of the poly(GMA-*co*-1,4DMB) copolymer (Figure 6A) in the initial step of pyrolysis was proposed. This mechanism leads to the emission of monomers, acrolein, carbon dioxide and the formation of unsaturated bonds in the solid residue.

**Figure 7:**
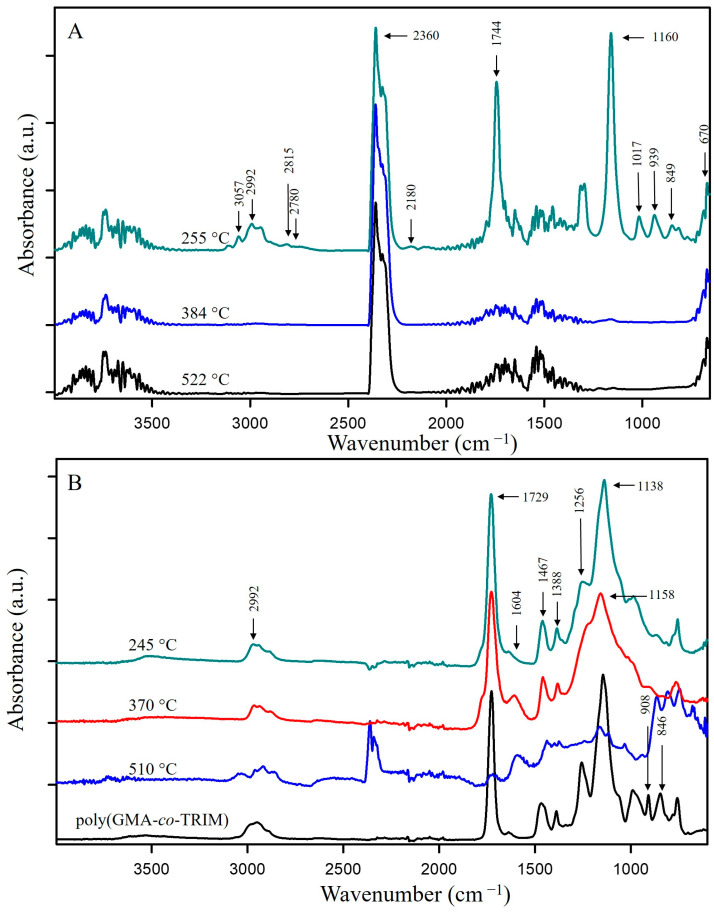
FTIR spectra of (**A**) gases released from poly(GMA-*co*-TRIM) at the maxima of emission; (**B**) preheated poly(GMA-*co*-TRIM).

In the FTIR spectrum of the gaseous poly(GMA-*co*-TRIM) decomposition products collected at the first emission maximum value at 255 °C (Figure 7A), bands characteristic of polyGMA decomposition by depolymerization can be observed. The absorption bands at 849 cm^−1^ associated with oxirane ring vibrations [41], those at 1160 cm^−1^ from C-O-C vibrations in the ester, those at 1744 cm^−1^ from vibrations of the C=O group and those at 3057 cm^−1^ related to the methylene group vibrations in the oxirane ring indicate the emission of GMA monomers [39,42]. For the TRIM-crosslinked copolymer, the bands characteristic of acrolein are not as intense as in the case of the copolymer with 1,4DMB, but bands in the spectrum region of 2700–2800 cm^−1^ can still be observed (Figure 5) [16]. Since an overall endothermic peak is observed in the DSC curve in the temperature region of the first degradation step, it can be assumed that the possible isomerization of oxirane groups is not as intense as in the case of the 1,4DMB-crosslinked copolymer and methacrolein is probably released due to the polymer network breakdown. In addition, at higher decomposition temperatures, mainly bands characteristic of water (3500–3600 and 1500–1600 cm^−1^), carbon dioxide (2360 cm^−1^) and carbon monoxide (2180 and 670 cm^−1^) formed by decarboxylation and dehydration processes of the created solid products of pyrolysis are observed [39,40,46]. Moreover, in order to follow the changes in the chemical structure of the copolymer during heating, it was heated to 245, 370 and 510 °C in a helium atmosphere, and then FTIR/ATR spectra of the preheated samples were recorded. These spectra were compared with the FTIR spectrum of the initial sample (Figure 7B). For the sample preheated to 245 °C, bands characteristic of oxirane ring vibrations at 846 and 908 cm^−1^ [41] and a band at 1256 cm^−1^ associated with oxirane ring stretching vibrations were not observed [45]. At 370 °C, a decrease in the intensity of the bands caused by the vibrations of the methyl and methylene groups (2965–3000 cm^−1^) and a shift in the band of C-O-C group vibrations from 1158 cm^−1^ to 1138 cm^−1^ can be observed [48]. Moreover, at 1604 cm^−1^, a new band appears indicating the formation of unsaturated C=C groups. Figure 6B proposes a decomposition scheme for the TRIM-crosslinked copolymer [47].

**Figure 8:**
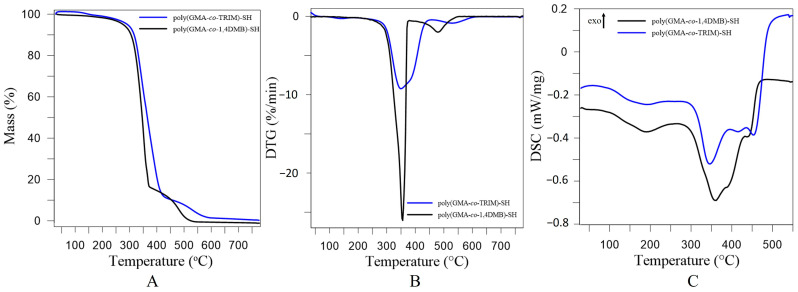
TG (**A**), DTG (**B**) and DSC (**C**) curves of poly(GMA*-co-*1,4DMB)-SH and poly(GMA*-co-*TRIM)-SH copolymers obtained under non-oxidative conditions.

TG, DTG and DSC curves for the studied microspheres after functionalization are presented in Figure 8, whereas the T_5%_, T_50%_ and T_max_ temperatures and the corresponding mass losses (Δm) are given in Table 1. From the DTG curves, it can be seen that both copolymers undergo a two-stage degradation process after functionalization. Each degradation stage is endothermic, which is confirmed by the DSC curves. The thermal resistance of the thioglycolic acid functionalized copolymers, inferred from T_5%_ values, increased compared to the initial copolymers by 54 °C for poly(GMA*-co-*TRIM)-SH and by 22 °C for poly(GMA*-co-*1,4DMB)-SH. The functionalized copolymers, compared to the initial ones, show no mass loss at around 250 °C (T_max1_), which is associated with the degradation of the structure fragments containing oxirane groups. Poly(GMA*-co-*TRIM)-SH reveals the first maximum value of degradation (T_max2_) at 344 °C and loses 89.2% of its mass at this stage (Δm_2_). The second maximum value (T_max3_) is observed at 531 °C with a mass loss of 9.5% (Δm_3_). Poly(GMA*-co-*TRIM)-SH has a higher thermal resistance by 16 °C in comparison with poly(GMA*-co-*1,4DMB)-SH. Regarding poly(GMA*-co-*1,4DMB)-SH, the first degradation maximum (T_max2_) occurs at 355 °C and is related to a mass loss of 83.9%. In contrast, the second maximum value (T_max3_) is observed at 479 °C with a mass loss of 16.6% (Δm_3_). In the case of functionalized copolymers, contrary to the initial copolymers, the TRIM-crosslinked copolymer has a higher thermal resistance. This may be due to the fact that this copolymer contained more oxirane groups in its structure before functionalization than the copolymer crosslinked with 1,4DMB, as evidenced by the higher value of the epoxide number.

**Figure 9:**
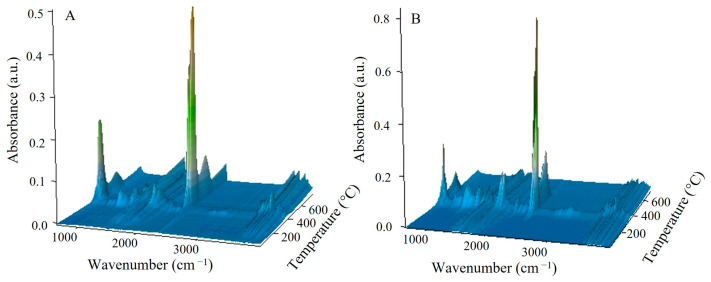
Three-dimensional FTIR diagrams of gaseous products of poly(GMA-*co*-TRIM)-SH (**A**) and poly(GMA-*co*-1,4DMB)-SH (**B**) degradation.

**Figure 10:**
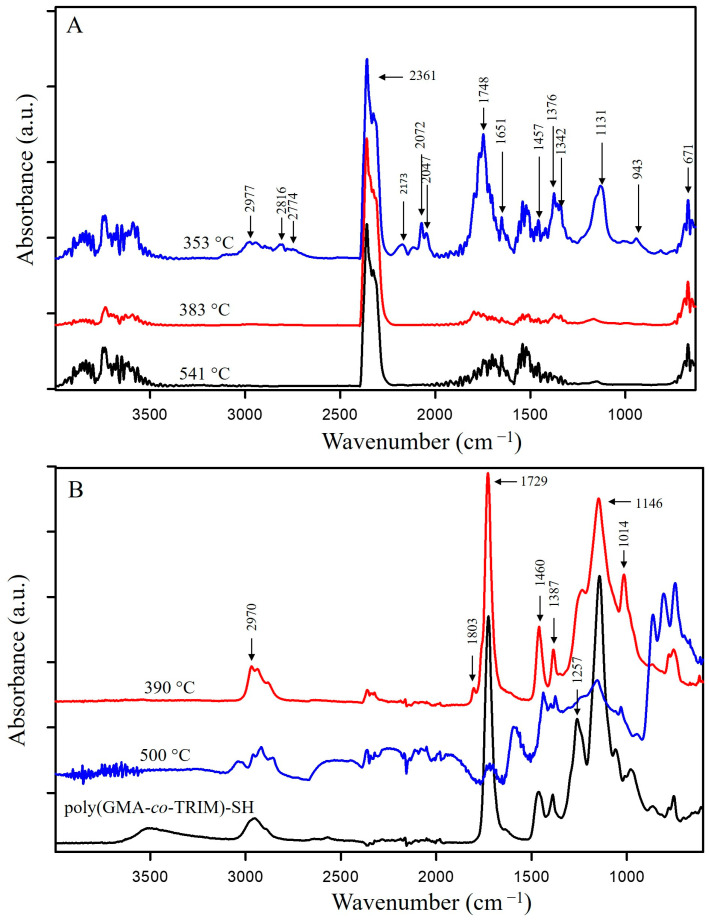
FTIR spectra of (**A**) gases released from poly(GMA-*co*-TRIM)-SH at the maxima of emission; (**B**) preheated poly(GMA-*co*-TRIM)-SH.

**Figure 11:**
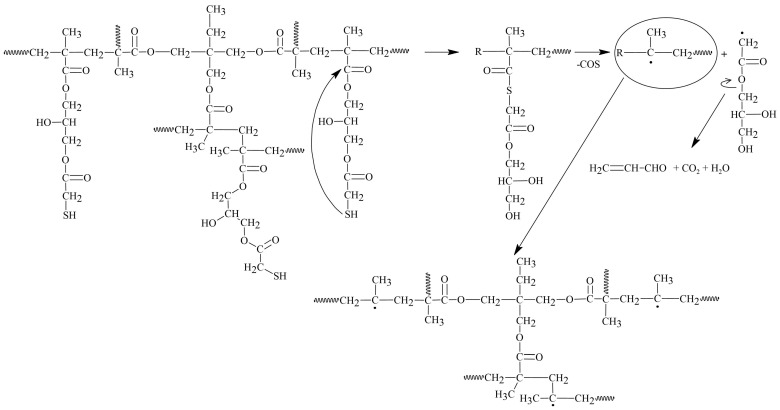
The possible decomposition path of poly(GMA-*co*-TRIM)-SH copolymer.

**Figure 12:**
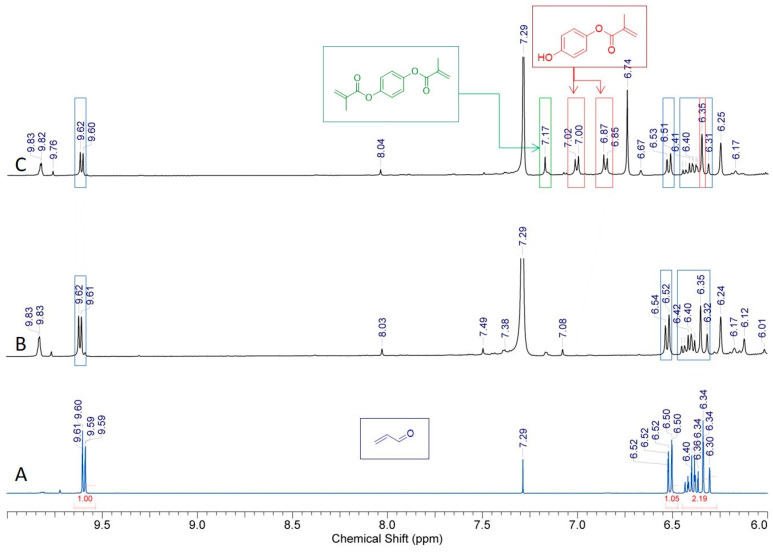
Excerpts of ^1^H NMR spectra of acrolein (**A**) and the substances that are produced during the thermal degradation of poly(GMA-*co*-TRIM)-SH (**B**) and poly(GMA-*co*-1,4DMB)-SH (**C**) recorded in CDCl_3_.

From the 3D-FTIR diagram of the gaseous pyrolysis products of poly(GMA*-co-*TRIM)-SH (Figure 9), the volatile spectra that evolved at the emission maxima were extracted. The spectrum collected at 353 °C (Figure 10A) shows absorption bands above 3500 cm^−1^ (the -OH stretching vibrations), at 3100 cm^−1^ (from the stretching vibrations of the =C-H) [39], in the range of 2970–2866 cm^−1^ (the C-H stretching vibrations), at 2806–2774 cm^−1^ (the aldehyde C-H stretching vibrations) [49], at 2360–2326 cm^−1^ (indicating CO_2_ presence), at 2173–2114 cm^−1^ (for CO) [39,40,46], at 2072–2047 cm^−1^ (representing the C=O vibration in carbonyl sulfide (COS) [50,51]), at 1790–1713 cm^−1^ (for the C=O stretching vibrations) [40], at 1651 cm^−1^ (for the C=C stretching vibrations), at 1457 and 1376–1342 cm^−1^ (for the C-H deformation vibrations), at 1131 cm^−1^ (for the C-O deformation vibrations) and at 943 cm^−1^ (for the =C-H out-of-plane deformation vibrations) [41]. Considering the 3D FTIR spectrum, it is evident that the maximum CO_2_ emission occurs between the first and second steps of copolymer degradation around 403 °C. In the spectrum from T_max3_, only the absorption bands derived from water (3600–3500 and 1500–1600 cm^−1^) and CO_2_ (2360–2326 and 671 cm^−1^) can be observed [49]. On the basis of the obtained FTIR results, the probable path of poly(GMA-*co*-TRIM)-SH copolymer degradation can be proposed (Figure 11). The presence of COS in the evolved gaseous mixture is the effect of the transesterification reaction between the thiol and carbonyl group followed by the rearrangement and finally the evolution of acrolein and carbon dioxide. ^1^H NMR analysis confirmed the evolution of acrolein in the first stage of pyrolysis (Figure 12A,B). Upon analyzing the solid residues after heating the samples to 390 and 500 °C, distinct differences compared to the non-heated sample spectrum can be observed (Figure 10B). First of all, in the spectra of preheated samples, there is a lack of absorption bands derived from the SH group vibrations (2641, 2569 cm^−1^). This confirms the evolution of COS at the beginning of the copolymer thermal degradation [50,51]. Additionally, in the spectrum region of the carbonyl stretching vibrations ( 390 °C), the new absorption band at 1803 cm^−1^ is visible and moreover, the main band is broadened with the shoulder at about 1760 cm^−1^. Furthermore, a significant new band at 1014 cm^−1^ (C-O-C) appears. These changes are most probably connected with the creation of lactone groups in the copolymer structure during heating [49].

**Figure 13:**
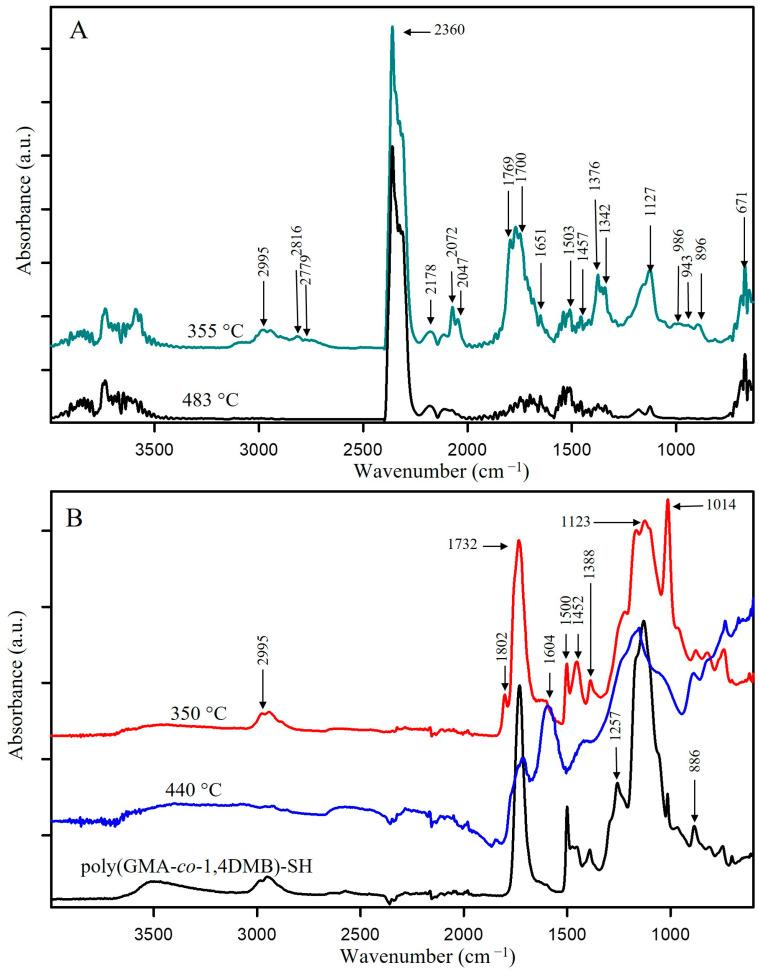
FTIR spectra of (**A**) gases released from poly(GMA-*co*-1,4DMB)-SH at the maxima of emission; (**B**) preheated poly(GMA-*co*-1,4DMB)-SH.

**Figure 14:**
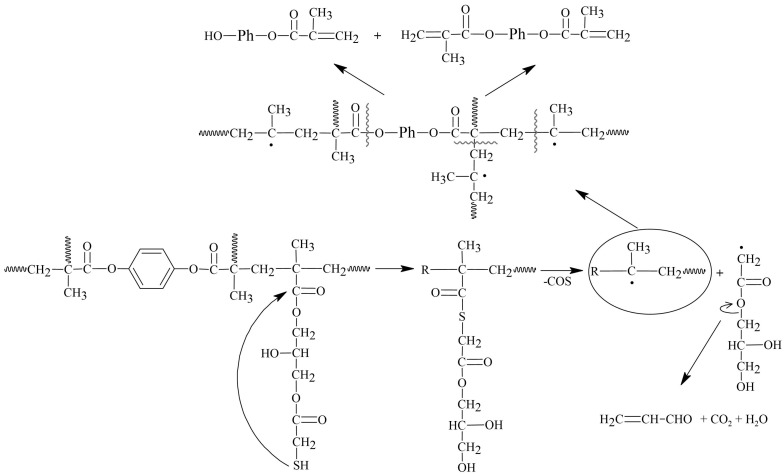
The possible decomposition path of poly(GMA-*co*-1,4DMB)-SH copolymer.

In the spectrum of gaseous products of poly(GMA-*co*-1,4DMB)-SH pyrolysis recorded at the first maximum of emission at 355 °C (Figure 13), the following absorption bands are visible: bands above 3500 cm^−1^ (the -OH stretching vibrations), at 3080 cm^−1^ (from the stretching vibrations of the =C-H) [39], in the region of 2980–2890 cm^−1^ (the C-H stretching vibrations), at 2816–2747 cm^−1^ (the aldehyde C-H stretching vibrations) [49], at 2360–2326 cm^−1^ (CO_2_), at 2173–2114 cm^−1^ (CO) [39,40,46], at 2072–2047 cm^−1^ (the C=O vibration in COS) [50,51], at 1794–1700 cm^−1^ (the C=O stretching vibrations) [40], at 1651 cm^−1^ (the C=C stretching vibrations), at 1503 cm^−1^ (the aromatic C=C stretching vibrations), at 1457 and 1376–1342 cm^−1^ (the C-H deformation vibrations), at 986 and 943 cm^−1^ (the =C-H out-of-plane deformation vibrations) and 896 cm^−1^ (the out-of-plane C-H vibrations of 1,4 substitute aromatic ring) [41]. The maximum carbon dioxide emission is observed at 370 °C, whereas in the spectra collected at 383 °C and 483 °C, the intensity of absorption bands derived from organic groups is smaller than at 355 °C. However, it is possible to clearly distinguish in these spectra bands at 1180, 1125, 946, 910 and 816 cm^−1^, which can be assigned to the presence of 1,4DMB derivatives. The veracity of these assumptions was confirmed by analyzing the ^1^H NMR spectrum of the compound’s mixture obtained from TG analysis up to 390 °C. The spectral data revealed the presence of signals corresponding to acrolein, 1,4DMB monomer, and a monomethacrylate derivative of hydroquinone, as depicted in Figure 12C. From the obtained spectral results, it can be assumed that the path of poly(GMA-*co*-1,4DMB)-SH copolymer thermal degradation is similar to the poly(GMA-*co*-TRIM)-SH. Figure 14 presents the probable course of poly(GMA-*co*-1,4DMB)-SH copolymer degradation. At the beginning, the transesterification of ester groups with -SH groups takes place, leading to the evolution of COS, acrolein and CO_2_. The maximum COS emission is observed at 342 °C, while the organic products are emitted with the highest intensity at 355 °C. This observation indicates that copolymer decomposition begins with transesterification. This process results in the creation of lactone groups in the solid residue as shown by bands at 1802 and 1014 cm^−1^ in the FTIR spectrum of the solid residue at 350 °C (Figure 13). Moreover, in this spectrum, the significant new band can be observed at 1604 cm^−1^, which is assigned to the C=C stretching vibrations. After disintegration of ester groups, the crosslinked aromatic skeleton is degraded, causing evolution of the 1,4DMB monomer and monomethacrylate hydroquinone. The additional advantage of using NMR analysis to identify the products formed during the thermal degradation of copolymers is the possibility of performing quantitative analysis. Based on proton signal integration, the ratio of hydroquinone monomethacrylate, 1,4DMB and acrolein was determined to be 4.7:1:9.2, respectively. Furthermore, it should be noted that the degradation of both thiol-containing copolymers is complicated due to their crosslinked structure making the identification of all degradation by-products impossible.

## 4. Conclusions

Novel copolymeric porous microspheres crosslinked with methacrylate comonomers and bearing thiol groups were characterized in terms of their behavior when heated in a non-oxidizing atmosphere. Analytical techniques such as DSC, TG/FTIR and ^1^H NMR were employed to investigate the impact of copolymer structure on thermal properties and degradation patterns. It was found that the modification reaction performed on the starting polymeric microspheres influenced the thermal stability and the degradation pathways of the studied materials. The incorporation into copolymer networks of thiol groups enhanced their thermal stability in comparison with the initial copolymers containing oxirane rings. The degradation of both starting copolymers involves three stages and commenced with depolymerization, yielding GMA and either acrolein or methacrolein. On the other hand, the degradation of thiol-functionalized copolymers took place in two steps. The emission of carbonyl sulfide at the beginning of the degradation suggests that the breakdown of the copolymeric networks is initiated by the transesterification reaction of the ester group with thiol and leads to the evolution of acrolein, while radical reactions cause the degradation of crosslinked networks. Through the application of ^1^H NMR analysis, mono- and dimethacrylate hydroquinone esters have been identified as the pyrolysis products of the poly(GMA-*co*-1,4DMB)-SH copolymer. Finally, one more issue needs to be mentioned. Since the studied copolymers have the potential to act as sorbents, the issue of their regeneration or disposal after the sorption process should be considered. The methods of sorbent regeneration include thermal desorption, whereas the spent polymer sorbents can be utilized in a carbonization process under a non-oxidative atmosphere and recycled into activated carbons. These two approaches to the treatment of used polymers require knowledge of their thermal resistance, as well as the qualitative composition of their degradation products.

## Figures and Tables

**Table 1:** TG and DTG data for the prepared microspheres.

Sample	T_5%_ (°C)	T_50%_ (°C)	T_max1_ (°C)	Δm_1_ (%)	T_max2_ (°C)	Δm_2_ (%)	T_max3_ (°C)	Δm_3_ (%)
poly(GMA-*co*-TRIM)	238	333	251	39.4	375	49.3	519	9.6
poly(GMA-*co*-TRIM)-SH	292	365	-	-	353	89.2	531	9.5
poly(GMA-*co*-1,4DMB)	254	346	269	33.8	388	49.9	556	11.2
poly(GMA-*co*-1,4DMB)-SH	276	346	-	-	355	83.9	479	16.6

## Data Availability

The data presented in this study are available upon request from the corresponding author.
